# Erratum: Stargardt macular dystrophy: common *ABCA4* mutations in South Africa—establishment of a rapid genetic test and relating risk to patients

**Published:** 2013-08-06

In this article by Lisa J. Roberts and colleagues (Molecular Vision v18:280-289), the following corrections were made:

A correction was made to Table 4, page 286. The numbering of the nucleotide change was corrected from "c.1885C>T" to "c.1804C>T".

